# Supplementary material for: Pseudodoping of a metallic two-dimensional material by the supporting substrate
Source: Nat Commun. 2019 Jan 14;10:180. doi: 10.1038/s41467-018-08088-8 (PMC6331619; doi:10.1038/s41467-018-08088-8)
Supplement: Supplementary file 1 — Supplementary Information [file 41467_2018_8088_MOESM1_ESM.pdf]

Supplementary Information

Pseudodoping of a Metallic Two-Dimensional Material by The Supporting Substrate

Shao et al

Supplementary Figures

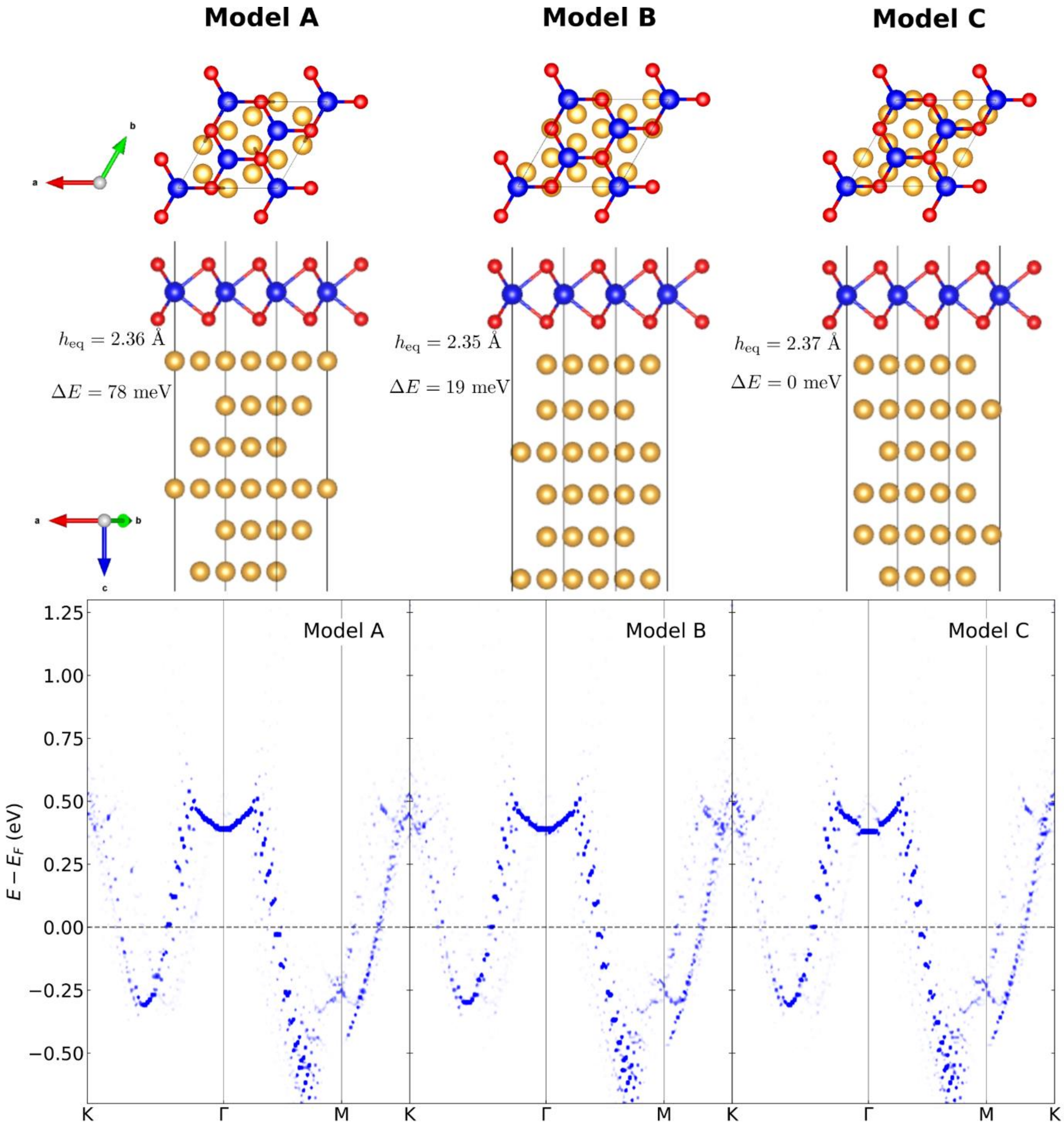

Supplementary Figure 1: Comparison of the results of three stacking arrangements of the TaS_2 (√3×√3)R30° supercell and the Au (111) 2×2 supercell. Upper panel: Top and side views of the three stacking arrangements. The Ta atoms, S atoms, and Au atoms are represented by blue, red, and yellow balls, respectively. The thickness of the Au slab in all the models is 30 atomic layers. The side of the Au slab without TaS_2 coverage was terminated by H atoms. A separation of more than 14 Å of vacuum between the periodically repeated slabs in the *c* direction was used to avoid the interaction between the successive surfaces. h_{eq} refers to the relaxed adsorption height. ΔE is the total energy of the given structure subtracted by that of "model C". Thus, the lowest energy structure belongs to "model C", which is used for the discussion in the main text. Bottom panel: the corresponding upfolded band structures, which depict states with Ta-*d* orbital character in terms of blue dots. It shows that the occupied parts of the upfolded band structures are virtually indistinguishable and there is hardly any influence of the exact stacking registry on the band shifts and hybridizations effects. I.e. the pseudodoping effect is essentially

independent of the exact stacking registry accounted for in the supercell calculations. The major quantity determining the strength of coupling between the TaS_2 and the Au(111) substrate states is the adsorption height.

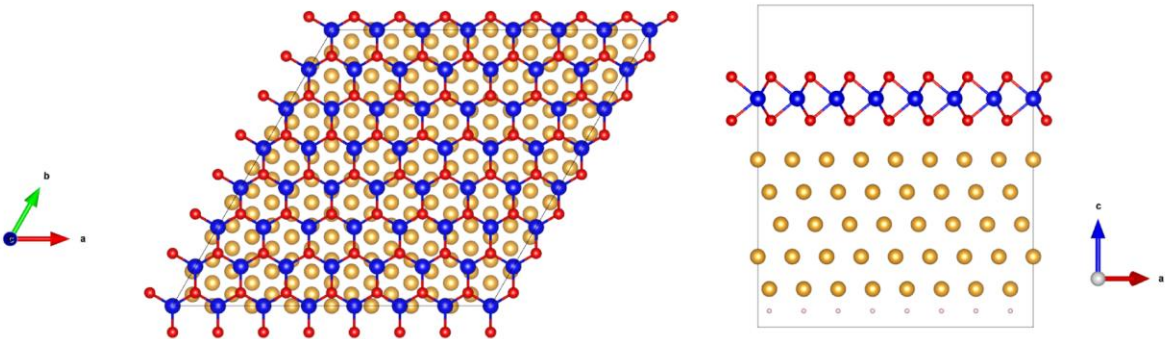

Supplementary Figure 2: Crystal structure of 7×7 supercell of TaS_2 on 8×8 supercell of Au(111). Left: top view. Right: side view. The Ta atoms, S atoms, Au atoms, and H atoms are represented by blue, red, yellow, and gray balls, respectively. The lattice constant of TaS_2 is 0.3317 nm, thus, the length of the 7×7 supercell of TaS_2 in *a*/*b* direction is about 2.3 nm, which is close to the moiré unit cell of the STM topography (< 2.5 nm). A thickness of 5 atomic layers slab was used for modeling the Au(111) substrate, which was terminated by H atoms on the bottom side. A separation of more than 6.5 Å of vacuum between the periodically repeated slabs in the *c* direction was used to avoid the interaction between the successive surfaces.

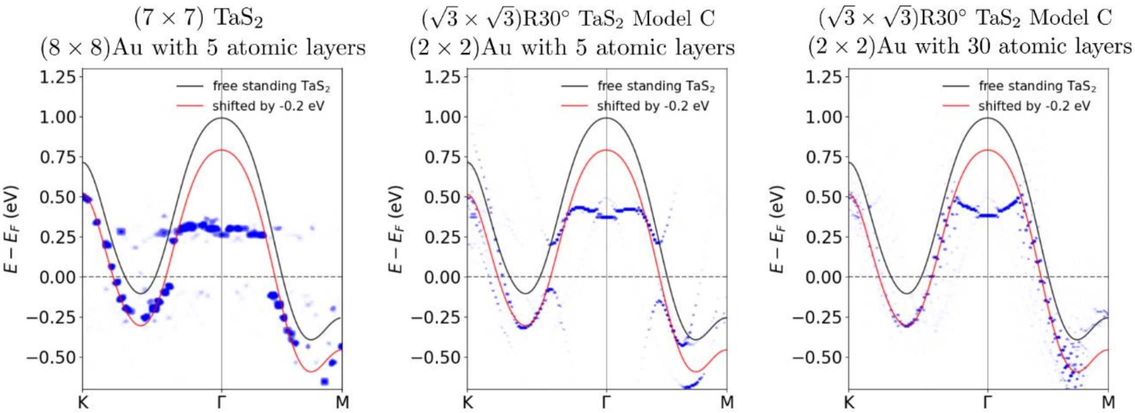

Supplementary Figure 3: Comparison of the upfolded band structures of 7×7 TaS_2 to (√3×√3)R30° TaS_2. For the 7×7 TaS_2 supercell (left panel), the adsorption height was set to that of the case in the middle panel (h_{eq} = 2.32 Å). The vacuum heights of the case in the left, middle, and right panel are >6.5 Å, >6.5 Å, and >14 Å, respectively. In the electronic structure calculations of the 7×7 TaS_2 supercell, we employed a 3×3×1 *k*-mesh sampling and a plane wave cut-off energy of 270 eV. For the case in the middle and right panel, we used a 21×21×1 *k*-mesh sampling and a plane wave cut-off energy of 400 eV.

Supplementary Tables

Type	Avg. peak energy	Std. dev.	Number of spots
No defect	373 mV	± 6 mV	20
Defect 1	431 mV	± 6 mV	7
Defect 2	455 mV	± 10 mV	11
Defect 3a	411 mV	± 17 mV	6
Defect 3b	512 mV	± 17 mV	6

Supplementary Table 1: Summary of statistical fluctuations in STS experiments. Each defect has been measured at as many spots as indicated in the column "number of spots" and we averaged over 5 spectra for each of the number of indicated spots in the table. The resulting average peaks and standard deviations in the peak positions are given in the second and third column, respectively.

Supplementary Notes

Supplementary Note 1: Influence of stacking registry between the TaS_2 (√3×√3)R30° supercell and the Au (111) 2×2 supercell on the pseudodoping. We have considered three possible stacking arrangements for the slab model of TaS_2 (√3×√3)R30° on the Au(111) 2×2 surface, as shown in Supplementary Figure1. We find very similar adsorption heights (differing only by < 0.02 Å) in all cases. The resulting upfolded band structures highlighting the bands with large Ta-*d* character are compared in the lower panel of Supplementary Figure 1. The occupied parts of the upfolded band structures are virtually indistinguishable and there is hardly any influence of the exact stacking registry on the band shifts and hybridizations effects. I.e. the pseudodoping effect is essentially independent of the exact stacking registry chosen in the supercell calculations. Indeed, the major quantity determining the strength of the coupling between the TaS_2 and the Au(111) substrate states is the adsorption height. Since the lowest energy stacking sequence belongs to geometry type "C" of Supplementary Figure1, all data we show in the main text correspond to this geometry.

Supplementary Note 2: Influence of the lateral supercell size on the pseudodoping. We have considered a 7×7 supercell of TaS_2 on 8×8 supercell of Au(111) which corresponds to the moiré unit cell observed in STM (see Supplementary Figure 2). Regardless of the lateral supercell size we find similar changes in the TaS_2 upfolded dispersion upon adsorption on Au (111): there is always a hybridization induced plateau around Γ and a downward shift of the occupied states on the order of -0.2eV (see Supplementary Figure 3). The major difference between the different supercell models arises from the different slab thicknesses. In the case of 5 Au layers quantum confinement leads to rather coarse quantization of the vertical crystal momentum k_z of the bulk Au conduction bands. Coupling of these k_z quantized Au bands to the TaS_2 conduction band leads to a few avoided crossing with apparent opening of minigaps in the TaS_2 which goes over into a broadening of the TaS_2 band in the case of thicker slabs (see model with 30 atomic Au layers) as it should be. Hence, we conclude that the thickness of the Au slab is the more critical parameter than the lateral extent of the supercell model. That is also in line with the finding of pseudodoping being mainly independent of the exact stacking realized at the interface of TaS_2 and Au(111), as can be seen from Supplementary Figure 1.
